# Empirical Research of Health Inequalities Between Male and Female Employees of the Hellenic National Organization for Healthcare Services Provision: A Cross-Sectional Study

**DOI:** 10.7759/cureus.85167

**Published:** 2025-06-01

**Authors:** Eleftheria Kouroukli, Evelina Pappa, Dimitrios Niakas

**Affiliations:** 1 Social Sciences, Hellenic Open University, Patras, GRC

**Keywords:** gender, health behavior, health inequalities, health-related quality of life, mental health, physical health

## Abstract

Aim

The study attempts to delineate a framework of investigation regarding health inequalities and health-related behaviors among male and female employees of the Hellenic National Organization for Healthcare Services Provision (EOPYY). The pillars of the presented framework are self-perceived health, the presence of chronic diseases, health behaviors, mental well-being, and healthcare utilization.

Subject and methods

A cross-sectional study was conducted on 209 employees using a self-administered online questionnaire. Data were analyzed using descriptive and inferential statistics (Mann-Whitney U, Kruskal-Wallis, as well as Chi-squared Χ²) and multiple linear regression analysis.

Results

Men reported better overall health, lower stress levels, and higher quality of life, particularly in dimensions such as physical pain (p=0.001) and mental health (p=0.002), whereas women reported a higher prevalence of chronic diseases and more frequent doctor visits. Women adopted healthier dietary habits and exercised more frequently, while men exhibited higher rates of overweight and obesity (p<0.001). Gender emerged as a significant predictor of both physical and mental health, with men demonstrating more favorable health indicators.

Conclusions

While health inequalities are gaining global attention, their impact on employees in large public Organizations in Greece remains underexplored. This study generates novel insights to shape strategies such as flexible work policies, improved workplace health programs, and gender equality education, driving a more inclusive and supportive work culture. Practical implications include workplace mental health support, targeted health promotion initiatives, and policies enhancing work-life balance. These measures can reduce gender-related health disparities and improve overall employee well-being and productivity. The findings may inform workplace health policies and promote gender-sensitive interventions in the public sector. The findings may inform workplace health policies and promote gender-sensitive interventions in the public sector.

## Introduction

Health is a fundamental factor for the well-being of employees and the productivity of an organization. However, health inequalities between men and women remain a significant challenge, influenced by biological, social, and psychological factors [1,2]. Workplaces, as microcosms of society, can reflect these differences more vividly as they are significantly associated with health inequalities [3] and therefore their study may contribute to the development of health equity policies [4].

The present study is grounded in the theoretical framework of the Social Determinants of Health (SDH), which posits that health outcomes are significantly influenced by the conditions in which individuals are born, grow, live, work, and age [5]. These determinants encompass factors such as socioeconomic status, education, employment, social support networks, and access to healthcare services. Addressing these social determinants is crucial for reducing health disparities and achieving health equity [6]. In the context of gender-based health inequalities, the SDH framework provides a lens through which to examine how societal structures and roles contribute to differential health outcomes between men and women. This approach is particularly relevant in the Greek public sector, where traditional gender roles and occupational segregation may influence health behaviors and access to healthcare resources.

Literature suggests that men and women experience different forms of occupational stress and have unequal access to healthcare services [7]. Women often bear a greater burden of family responsibilities, while men may hesitate to express mental health issues due to social stereotypes [8]. These differences can impact quality of life, job performance, and healthcare utilization.

The aim of this study is to investigate health inequalities and health-related behaviors among male and female employees of the Hellenic National Organization for Healthcare Services Provision (EOPYY). Through the analysis of data collected via a self-administered questionnaire, the study aims to identify differences in physical and mental health, healthcare utilization, and daily behaviors that influence employee well-being.

The National Organization for the Provision of Health Services (EOPYY) is Greece's primary public health insurance provider. Established in 2011, it unified the healthcare benefits previously managed by multiple social security funds. EOPYY's main role is to ensure access to high-quality healthcare services for citizens in both the public and private sectors, through a network of contracted doctors, hospitals, diagnostic centers, and pharmacies. It is primarily funded by employee and employer contributions, along with state resources, and covers a wide range of medical services, including primary, secondary, and tertiary care, pharmaceutical treatments, and diagnostic tests for insured individuals.

Although health inequalities have gained increasing international attention, empirical evidence on their impact within large public sector organizations in Greece is scarce. Most existing studies focus on the general population or specific patient groups, leaving a gap in understanding how gender-related disparities manifest in workplace settings. This study seeks to fill this gap by focusing on employees of EOPYY, the largest public health insurance organization in the country. Investigating these inequalities provides evidence-based insights for the development of strategies such as flexible work schedules, the enhancement of workplace health support programs, and the promotion of education for awareness on gender equality issues. These initiatives contribute to fostering a culture of respect, empowerment, and inclusion.

## Materials and methods

Study design

A cross-sectional study was conducted by using a questionnaire in order to investigate health inequalities and health-related behaviors among male and female employees of EOPYY.

Cross-sectional studies provide a snapshot of the variables under investigation at a specific point in time, allowing the identification of associations but not causal relationships [9]. Moreover, they are relatively quick and inexpensive to conduct.

Sample and sampling procedure

The study involved EOPYY employees from the Central Administration of EOPYY, who were invited via email to voluntarily complete an anonymous online questionnaire (Google Forms; Google, Mountain View, California). No access to individual email addresses was granted, as the invitation was sent to the group emails of the Organization's Directorates.

A total of 300 employees were invited to participate, including 226 women (75.33% of the total) and 74 men (24.67% of the total). Ultimately, 209 employees (69.67% of all invited employees) responded, comprising 158 women (75.60% of the sample) and 51 men (24.40% of the sample).

The convenience sampling approach allows for efficient and cost-effective data collection; however, this method may restrict the extent to which the findings can be generalized [10].

Research instrument

The questionnaire included the following sections: demographic information (gender, age, height, weight, marital status, education level, income, household size); health status (doctor visits in the past month, presence and number of chronic diseases, hospitalizations in the past year); health behaviors (smoking, alcohol consumption, dietary habits (consumption of fast food, fruit, and vegetables), frequency of physical activity, sleep quality); quality of life (the Greek version of the Short Form-12 Health Survey (SF-12) was used to assess health-related quality of life (HRQoL), measuring both the Physical Component Score (PCS) and Mental Component Score (MCS) [11, 12])

Ethical considerations

The study was conducted in accordance with ethical research principles. Participants were informed about the study's purpose and participated voluntarily. Data collection was conducted anonymously to ensure confidentiality.

Data analysis

The collected questionnaire data were coded and analyzed using SPSS software version 29.0.2.0 (IBM Inc., Armonk, New York). The statistical analysis included descriptive analysis and inferential analysis. Frequencies and percentages were calculated for categorical variables, while means, medians, and standard deviations were reported for continuous variables. Non-parametric tests, including the Mann-Whitney U and Kruskal-Wallis tests, were used to examine differences between men and women regarding health status, health behaviors, and quality of life. The Chi-squared test of independence (Χ²) was applied to assess relationships between categorical variables.

Multiple linear regression was used to examine the impact of socio-demographic factors and health behaviors on health-related quality of life (HRQoL). Model fit was evaluated using the coefficient of determination (R²), the F-test, and t-tests for the statistical significance of independent variables.

All statistical analyses were conducted at a significance level of α = 0.05.

## Results

Table 1 presents the demographic and socioeconomic characteristics of the 209 EOPYY employees who participated in the study. The sample predominantly consists of women (n=158, 75.6%), with the most common age group being 46-55 years (n=121, 57.9%). Most participants are married (n=151, 72.2%) and hold a higher education or postgraduate degree (n=159, 76%). Regarding the financial profile, n=147 (70.3%) of households have a monthly income between €1000 and €3000.

**Table 1:** Demographic and socioeconomic characteristics of the participants

		n	Percentage
Gender	Male	51	24.4%
	Female	158	75.6%
Age	25-35	4	1.9%
	36-45	37	17.7%
	46-55	121	57.9%
	56+	47	22.5%
Marital Status	Single	37	17.7%
	Married	151	72.2%
	Divorced	15	7.2%
	Widowed	6	2.9%
Education level	Secondary school	1	0.5%
	High school	24	11.5%
	Post-secondary education	19	9.1%
	University fegree	86	41.1%
	Master's degree	73	34.9%
	Doctorate	6	2.9%
Household size (number of persons)	1	23	11.0%
	2	44	21.1%
	3	49	23.4%
	4	75	35.9%
	5	14	6.7%
	6	4	1.9%
Monthly household income	Up to 1000€	3	1.4%
	1001€-2000€	68	32.5%
	2001€-3000€	79	37.8%
	Above 3000€	59	28.2%

Table 2 presents the differences in health status between men and women. Significantly higher rates of overweight and obesity were observed among men (p<0.001), while women were more likely to maintain a normal body weight. Women also reported more frequent doctor visits (p=0.020), although no statistically significant differences were found in hospitalization rates or the diagnosis of chronic diseases. However, women were more likely to report multiple chronic conditions, with n=27 (17%) suffering from three or more chronic diseases, whereas the corresponding percentage among men was zero (n=0, 0%).

**Table 2:** Health status by gender

		Gender		Χ^2^	p-value
		Male	Female		
Body mass index (BMI)	Underweight	0.0%	2.5%	21.552	0.000
	Normal weight	15.7%	50.0%		
	Overweight	47.1%	25.3%		
	Obese	37.3%	22.2%		
Have you visited a doctor in the past month?	No	51.0%	32.9%	5.381	0.020
	Yes	49.0%	67.1%		
Have you been hospitalized in the past year?	No	96.1%	90.5%	1.602	0.206
	Yes	3.9%	9.5%		
Do you suffer from a chronic disease?	No	72.5%	66.5%	0.657	0.418
	Yes	27.5%	33.5%		
Number of chronic diseases	1	64.3%	54.7%	2.760	0.252
	2	35.7%	28.3%		
	3 or more	0.0%	17.0%		

Regarding health behaviors between men and women, no statistically significant differences were observed in alcohol consumption, smoking, exercise frequency, or sleep quality. However, women consumed significantly more fruit and vegetables compared to men (p=0.003), while men showed slightly higher rates of fast-food consumption.

Table 3 shows that men had a significantly higher score in the mental health dimension of quality of life (MCS12, p=0.038), while no statistically significant difference was observed between the two genders in the physical health dimension (PCS12, p=0.066).

**Table 3:** Comparison of quality of life between men and women PCS12 - Physical Component Score, MCS12 - Mental Component Score, M - mean, SD - standard deviation, Md - median, U - Mann-Whitney U test (U)

		Gender					U	p-value
	Male			Female				
	M	SD	Md	M	SD	Md		
PCS12	50.0	10.2	55.1	48.3	9.7	52.1	3338.0	0.066
MCS12	48.9	8.5	50.4	45.2	10.6	46.5	3248.0	0.038

The results of the multiple linear regression model for the physical health dimension (PCS) indicate that higher BMI, doctor visits in the past month, hospitalization in the past year, and the presence of a chronic disease predict worse health-related quality of life in terms of physical health (Table 4). In contrast, male gender, higher education level, and increased exercise frequency predict better health-related quality of life in terms of physical health.

**Table 4:** Multiple linear regression – significant predictors of health-related quality of life (PCS12) B - unstandardized coefficients, SD - standard deviation, Beta - standardized coefficients

Variable	B	SD	Beta	t-test	p-value
Gender	-3.369	1.656	-0.148	-2.035	0.043
BMI	-0.140	0.041	-0.247	-3.447	0.001
Education level	1.715	0.611	0.180	2.806	0.006
Have you visited a doctor in the past month?	-3.361	1.229	-0.166	-2.736	0.007
Have you been hospitalized in the past year?	-5.449	2.137	-0.152	-2.549	0.012
Do you suffer from a chronic disease?	-3.186	1.254	-0.152	-2.542	0.012
How often do you exercise?	3.594	0.679	0.342	5.290	0.000

Note: The overall model was statistically significant (F=7.596, p<0.001), explaining 37.1% of the variance in PCS12 (R²=0.371).

The results of the multiple linear regression model for the mental health dimension (MCS) identified gender and exercise frequency as the only statistically significant predictors of health-related quality of life in terms of mental health (Table 5). Specifically, women had lower mental health scores (B=-4.955, p = 0.016), while more frequent exercise was associated with higher mental well-being (B=1.876, p=0.026). The overall model was statistically significant (F=1.771, p=0.041) but explained only 12.1% of the variance in MCS12 (R²=0.121). This indicates a limited explanatory power and suggests that key determinants of mental health, potentially including psychosocial, occupational, or environmental factors, were not captured in this model. These findings should therefore be interpreted with caution, and future studies are needed to further explore the broader determinants of mental well-being in workplace contexts.

**Table 5:** Multiple linear regression – significant predictors of health-related quality of life (MCS12) B - unstandardized coefficients, SD - standard deviation, Beta - standardized coefficients, MCS12 - Mental Component Score

Variable	B	Std. Error	Beta	t-test	p-value
Gender	-4.955	2.037	-0.209	-2.433	0.016
How often do you exercise?	1.876	0.836	0.172	2.244	0.026

## Discussion

The findings of this study highlight significant differences between male and female employees of EOPYY regarding health behavior, physical and mental health, and the use of healthcare services. These results align with previous studies on gender health disparities and emphasize the need for tailored health policies that consider the distinct needs of both genders.

Differences in health behavior

Male EOPYY employees exhibited higher rates of increased body weight and obesity compared to their female counterparts, confirming prior research indicating that men consume more calories from sources rich in fats and proteins [13,14]. However, obesity appears to have a greater impact on women's mental health, with female employees reporting higher levels of emotional distress and lower self-esteem.

Additionally, female employees reported higher consumption of fruit and vegetables, a finding which is consistent with literature suggesting that women prefer diets rich in fiber. Furthermore, higher educational attainment is associated with healthier dietary choices [15,16]. Similar gender differences were observed in physical activity, with women engaging in exercise more frequently, which is linked to better quality of life [13]. However, the literature indicates that men tend to choose different forms of physical activity, focusing more on muscle strengthening [17].

Differences in physical health

While the initial analysis of the SF-12 dimensions did not reveal statistically significant gender differences in the physical health component of quality of life, multiple linear regression analysis identified gender as an independent predictor, with men displaying slightly better physical health outcomes. This difference may reflect variations in lifestyle and social conditions that affect health [18,19].

An interesting finding concerns pain perception, with men reporting that pain did not significantly affect their work performance. The literature suggests that men may downplay or avoid reporting pain due to social norms related to "resilience" [20].

Use of healthcare services and chronic diseases

According to the study results, women visit doctors more frequently and report higher rates of chronic illnesses. Similar findings have been reported in international literature, indicating that women are more likely to seek medical care and report health problems [21,22]. Women use primary healthcare services more often, particularly for preventive purposes, whereas men tend to visit doctors only in cases of severe health issues [23].

Differences in mental health and quality of life

One of the most significant findings of the study concerns mental health, with male EOPYY employees scoring significantly higher compared to their female counterparts. Women reported greater emotional distress, suggesting a higher burden from stressors such as balancing work and family responsibilities [7]. Women are more likely to use emotion-focused coping strategies, which may contribute to increased psychological strain [24].

This observation is supported by studies showing that women exhibit higher levels of anxiety and depression, while men tend to avoid seeking psychological support unless they perceive their problem as severe [25]. Additionally, research has shown that socioeconomic inequalities negatively impact women's mental health [26]. These gendered patterns may reflect broader societal expectations in Greece, where traditional gender roles still influence emotional expression, caregiving responsibilities, and attitudes toward seeking professional support.

Intervention proposals

The study findings reinforce the need for targeted health interventions that address the different needs of men and women. Improving access to mental health services, promoting healthy eating habits, and enhancing physical activity programs can contribute to reducing health disparities.

Special attention should be given to adopting policies that support work-life balance, as increased stress among women is associated with their efforts to juggle multiple roles. Moreover, gender-sensitive health policies are necessary, especially concerning the prevention and management of chronic diseases [27].

This study serves as an initial attempt to capture gender health disparities in the Greek public sector. The results may be used by EOPYY to develop targeted health programs for employees, aiming at improving quality of life and promoting workplace well-being.

Study limitations

This study has certain limitations. The convenience sample (209 employees) may pose a risk of bias, although the gender distribution of the sample (75% women and 25% men) reflects the actual composition of EOPYY personnel. However, the use of a non-random sampling method and the focus on a single public sector institution limit the generalizability of the findings to other settings or populations. Since the collection of data was based on a self-administered questionnaire, the data are prone to response bias [28], particularly regarding sensitive issues such as emotional state and mental health. Despite these limitations, the study provides valuable insights into gender-based health inequalities in the workplace and lays the groundwork for broader investigations in the Greek public sector.

## Conclusions

The present study revealed notable gender-based differences in health perception, chronic disease prevalence, health behaviors, mental well-being, and healthcare service utilization. Men generally rated their health more positively and reported better quality of life in areas such as physical pain and mental well-being, whereas women reported higher rates of chronic conditions and a greater mental health burden, including elevated anxiety and emotional distress. Despite this, women were more likely to engage in health-promoting behaviors, such as more frequent consumption of fruit and vegetables and increased physical activity. No significant gender differences were found in smoking, alcohol consumption, or sleep quality. Additionally, women were more likely to use healthcare services, reflecting both heightened health needs and a greater tendency to seek preventive care.

The study suggests that health inequalities are shaped by both biological and sociocultural determinants, highlighting the potential value of personalized and gender-sensitive approaches in healthcare provision. Targeted interventions, such as gender-informed health promotion strategies, workplace mental health initiatives, and tailored service delivery, may contribute to improved overall employee well-being. Future research should adopt longitudinal and multicentric approaches to explore not only demographic and behavioral disparities but also the broader occupational, social, and economic influences on health. A deeper understanding of these factors is essential for developing effective workplace policies and fostering equity and sustainable development in the public sector. A brief qualitative follow-up could complement these findings by exploring the lived experiences behind the observed gender-related trends.

## Appendices

Survey Questionnaire

DEMOGRAPHIC AND ECONOMIC INFORMATION

1. Gender *

  Male   Female

2. Age *

  A. 25 - 35   B. 36 - 45   C. 46 - 55   D. 56+

3. What is your height (e.g., 182 cm)? *

4. What is your weight (e.g., 85 kg)? *

5. Marital status *

  A. Single   B. Married   C. Divorced   D. Widowed

6. Education level * (Select the highest level of education completed)

 A. Junior High School   B. High School   C. Post-secondary Education   D. University/College   E. Master's Degree   F. Doctorate

7. How many people are in your household? *

  Please enter the number

8. What is your household's total monthly income? *

  A. up to €1,000   B. €1,001 - €2,000   C. €2,001 - €3,000   D. over €3,000

9. Have you visited a doctor in the last month? *

  YES   NO

10. Have you been hospitalized in the last year? *

  YES   NO

11. Do you suffer from any chronic disease? *

  A. Yes -> Proceed to question 12   B. No -> Proceed to question 13

12. How many chronic diseases do you suffer from? *

  A. 1   B. 2   C. 3   D. more than 3

DAILY HABITS

13. How often do you consume alcohol? *

  A. Daily   B. 1-3 times a week   C. A few times a month   D. Never

14. Are you a smoker? *

  A. Regular smoker -> Proceed to question 15

  B. Occasional smoker -> Proceed to question 15

  C. Former smoker -> Proceed to question 16

  D. Never smoked -> Proceed to question 16

15. How many cigarettes do you smoke per day? *

  A. 1 - 10   B. 11 - 20   C. 21 - 40   D. More than 40

16. How often do you eat fast food? *

  A. Daily   B. 1 - 3 times a week   C. A few times a month   D. Never

17. How often do you consume fruits and vegetables? *

  A. Daily   B. 1 - 3 times a week   C. A few times a month   D. Never

18. How often do you exercise? *

  A. Daily   B. 1 - 3 times a week   C. A few times a month   D. Never

19. How would you describe your sleep quality? *

  A. Sleep deprivation   B. I sleep adequately

SF-12 QUESTIONNAIRE

20. In general, would you say your health is: *

  Excellent   Very good   Good   Fair   Poor

21. The following statements involve activities you might do during a typical day. Does your current health limit you in these activities? If yes, how much? *

a. Moderate activities, such as moving a table, using a vacuum cleaner, walking outdoors, or playing beach paddle

  Yes, limited a lot   Yes, limited a little   No, not limited at all

b. Climbing several flights of stairs

  Yes, limited a lot   Yes, limited a little   No, not limited at all

22. During the past 4 weeks, have you had any of the following problems in your work or daily activities due to your physical health? *

a. Accomplished less than you would like?   YES   NO

b. Were limited in the kind of work or other activities?   YES   NO

23. During the past 4 weeks, have you had any of the following problems due to emotional issues (e.g., feeling depressed or anxious)? *

a. Accomplished less than you would like?   YES   NO

b. Did work or activities less carefully than usual?   YES   NO

24. During the past 4 weeks, how much did pain interfere with your normal work (both outside the home and at home)? *

  Not at all   A little bit   Moderately   Quite a bit   Extremely

25. The following questions ask about how you felt and your mood in general over the past 4 weeks. Please answer as accurately as possible. *

a. How often have you felt calm and peaceful?

  All of the time   Most of the time   A good bit of the time   Some of the time   A little of the time   None of the time

b. Did you have a lot of energy?

  All of the time   Most of the time   A good bit of the time   Some of the time   A little of the time   None of the time

c. Have you felt downhearted and depressed?

  All of the time   Most of the time   A good bit of the time   Some of the time   A little of the time   None of the time

26. During the past 4 weeks, how much of the time has your physical health or emotional problems interfered with your social activities (like visiting friends or relatives)? *

  All of the time   Most of the time   A good bit of the time   Some of the time   A little of the time   None of the time
